# Clustered mutations in hominid genome evolution are consistent with APOBEC3G enzymatic activity

**DOI:** 10.1101/gr.199240.115

**Published:** 2016-05

**Authors:** Yishay Pinto, Orshay Gabay, Leonardo Arbiza, Aaron J. Sams, Alon Keinan, Erez Y. Levanon

**Affiliations:** 1 Mina and Everard Goodman Faculty of Life Sciences, Bar-Ilan University, Ramat Gan 5290002, Israel;; 2 Department of Biological Statistics and Computational Biology, Cornell University, Ithaca, New York 14853, USA

## Abstract

The gradual accumulation of mutations by any of a number of mutational processes is a major driving force of divergence and evolution. Here, we investigate a potentially novel mutational process that is based on the activity of members of the AID/APOBEC family of deaminases. This gene family has been recently shown to introduce—in multiple types of cancer—enzyme-induced clusters of co-occurring somatic mutations caused by cytosine deamination. Going beyond somatic mutations, we hypothesized that APOBEC3—following its rapid expansion in primates—can introduce unique germline mutation clusters that can play a role in primate evolution. In this study, we tested this hypothesis by performing a comprehensive comparative genomic screen for APOBEC3-induced mutagenesis patterns across different hominids. We detected thousands of mutation clusters introduced along primate evolution which exhibit features that strongly fit the known patterns of APOBEC3G mutagenesis. These results suggest that APOBEC3G-induced mutations have contributed to the evolution of all genomes we studied. This is the first indication of site-directed, enzyme-induced genome evolution, which played a role in the evolution of both modern and archaic humans. This novel mutational mechanism exhibits several unique features, such as its higher tendency to mutate transcribed regions and regulatory elements and its ability to generate clusters of concurrent point mutations that all occur in a single generation. Our discovery demonstrates the exaptation of an anti-viral mechanism as a new source of genomic variation in hominids with a strong potential for functional consequences.

Molecular evolutionary theory posits that diversification generally proceeds through the gradual accumulation of genetic variation, with point mutations as a prominent source of genetic heterogeneity within and among species. Mutations can be generated by spontaneous chemical reactions, be induced by mutagenic agents, or result from error-prone replication and repair mechanisms, among other processes. Such mutations are mostly assumed to occur randomly across the genome and, consecutively, can be targeted by natural selection. Although some exceptions have been described (Harris and Nielsen 2014; Zhu et al. 2015), the majority of single-nucleotide mutations are assumed to occur independently from each other (Hodgkinson and Eyre-Walker 2011; Campbell and Eichler 2013; Ségurel et al. 2014). These point mutations are considered to accumulate more or less steadily along evolutionary time, thereby acting as a molecular clock.

Similar to evolution driven by germline mutations, somatic mutations follow the same molecular mechanisms and accumulate over time (Simpson 1997) in a process that is considered to be the main driver for cancer formation. Recently, studies analyzing cancer genomes described the first evidence of enzyme-induced somatic mutations (Nik-Zainal et al. 2012; Roberts et al. 2012, 2013; Alexandrov et al. 2013; Burns et al. 2013a,b). These mutations are catalyzed by members of the vertebrate-specific AID/APOBEC family of deaminases, which introduce clusters of concurrent somatic mutations caused by cytosine deamination (Suspène et al. 2011; Caval et al. 2014). This phenomenon has been described in multiple cancer types, and enrichment of APOBEC-driven mutations has tied APOBEC catalytic activity to carcinogenesis (Burns et al. 2013b; Roberts et al. 2013; Henderson et al. 2014; Saraconi et al. 2014). Somatic mutagenesis is a novel function of AID/APOBEC proteins which were canonically documented as hypermutators of immunoglobulin genes (Teng and Papavasiliou 2007; Jaszczur et al. 2013) and act as anti-viral agents by mutating viral genomes (Kinomoto et al. 2007; Chiu and Greene 2008). These enzymes have also been observed to similarly introduce mutations to endogenous retroelements prior to their reintegration into the genome (Carmi et al. 2011). An additional study demonstrated that A3G mutagenesis within retroelements is inherited and may diversify the repertoire of retroelements during evolution (Knisbacher and Levanon 2016).

The evolution of the distinct paralogs in the *AID/APOBEC* family had been initiated from an ancestral *AID* gene that originally emerged in Vertebrata (Sawyer et al. 2004). In several different lineages, *APOBEC* genes were subjected to duplications and fusions, likely driven by selective pressures. One notable example is that of the *APOBEC3* (*A3*) gene, which in primates has expanded through duplication into a tandem array with seven different paralogs (*A3A*, *A3B*, *A3C*, *A3D*, *A3F*, *A3G*, and *A3H*) (Conticello 2008). It is broadly accepted that this expansion selectively emerged to cope with the rapid diversification of primate-specific retroelements and viruses (Sawyer et al. 2004; Zhang and Webb 2004).

Different A3 paralogs are known to have distinct preferences for the substrate nucleotide sequences on which they operate (Liddament et al. 2004; Shen and Storb 2004; Hultquist et al. 2011; Roberts et al. 2013). These paralogs all catalyze the deamination of cytosine to uracil on single-stranded DNA (ssDNA) within their specific sequence motif. While targeting genomic DNA, the cytosine deamination results in C-to-T/G/A substitutions due to replication or error-prone repair of the mutated DNA strand (Roberts et al. 2013).

Considering the rapid emergence of the different A3 enzymes in primates, and the fact that most of them are expressed in the germline (Koning et al. 2009; Refsland et al. 2010), we hypothesized that A3 can introduce mutation clusters in the germline and may have therefore played a role in primate evolution. This hypothesis is in contrast to an underlying principle of molecular evolution that point mutations occur independently of each other. Rather, the enzymatic activity of A3 may encompass more than one cytosine residue in the same locus and generate a cluster of concurrent point mutations, which all occur in a single generation. In this study, we sought to test for evidence of enzyme-induced evolution associated with APOBEC activity in the germline as was outlined previously (Harris et al. 2002). For this purpose, we performed a comprehensive comparative genomic screen for A3-induced mutagenesis patterns across different primates.

## Results

### Evidence for APOBEC3G activity in human evolution

**Figure 1.**
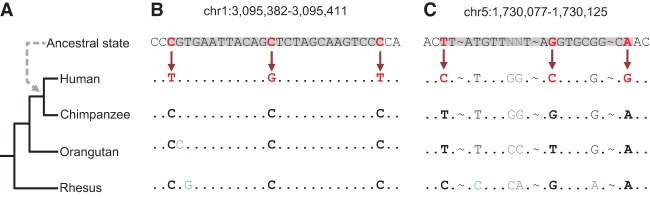
Clustered mutations in the human lineage. (*A*) Phylogenetic tree of the species used for computing the human-chimpanzee ancestral state (dashed arrow). (*B*) Multiple sequence alignment demonstrating an incidence of a C-coordinated cluster in which the ancestral state harbors closely located cytosine residues that were mutated in the human lineage. (*C*) An example of an N-coordinated cluster where the mismatches originated from different ancestral nucleotides. Alignment matches are represented by dots; omitted parts of the sequence are indicated by tildes (∼). Red letters and arrows indicate mismatches in the cluster. Positions in which an outgroup does not agree with the other outgroups are marked in blue, and positions in which the ancestral state could not be inferred are marked in gray. The boundaries of clusters are highlighted in gray.

We set out to investigate whether A3 proteins have led to enzyme-induced evolutionary changes via introduction of mutation clusters in the germline. For this purpose, we used an approach similar to that established for the detection of A3 activity in human cancer (Roberts et al. 2013). Briefly, the methodology exploits the fact that different A3 paralogs have a unique mutagenesis pattern that consists of preferred substrate nucleotide motifs and a tendency to mutate several cytosines on the same ssDNA strand (Supplemental Fig. S1). We first conducted a multispecies comparative genomic analysis that identified mutations that have occurred along the human lineage since its divergence from chimpanzee, i.e., positions in the reference human genome that differ from their ancestral state (Table 1; Fig. 1A; Methods). Second, we considered clusters of such mutations as groups of at least two mutations within ≤50 base pairs (bp) from each other (Fig. 1B,C). We excluded clusters with potential complex mutations that are ≤10 bp from each other and clusters composed of mutations arising from different DNA strands or different nucleotides in the ancestral state (Fig. 1C). To further minimize the extent to which nearby yet independently occurring mutations are considered as a cluster, we further excluded clusters according to a cluster specific *P*-value that accounts for the probability of clustering independent events (Roberts et al. 2012). We refer to these clusters by their ancestral nucleotide, e.g., a cluster with all mutations originating from cytosine is termed a C-coordinated cluster (Fig. 1B).

**Table 1.**
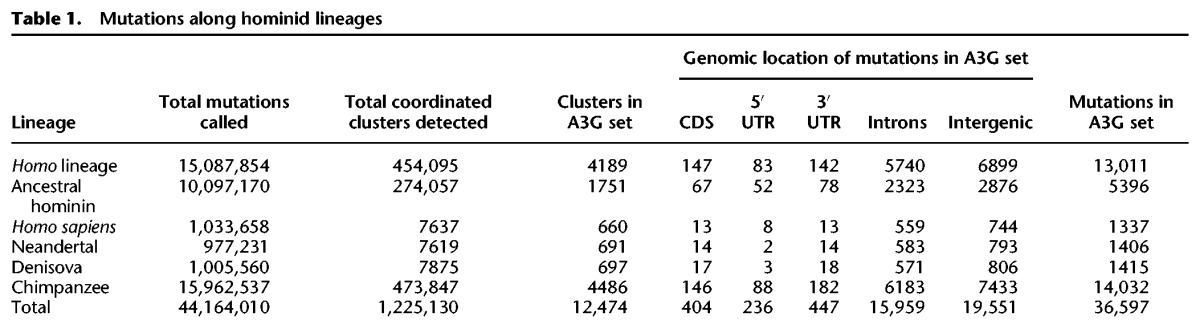
Mutations along hominid lineages

**Figure 2.**
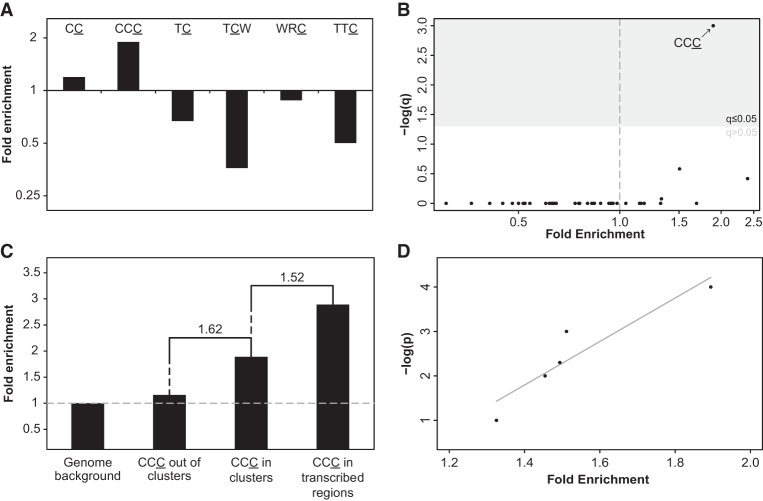
Identification of APOBEC3G mutagenesis pattern in human evolution. (*A*) A3G CC and CCC motifs are significantly enriched within the set of C- (or G-) coordinated clusters with cluster *P*-value ≤0.0001 (*q* < 0.006, one-tailed Fisher's exact test after Bonferroni correction. *P*-values were calculated by comparing the frequency of mutations within and outside a given motif while controlling for the frequency of C or G nucleotides within and outside this motif in the genome). Clusters are not enriched with any other APOBEC-related motif. (*B*) CCC, the A3G stringent motif, is the only CpG-free trinucleotide motif that is significantly enriched in C- (or G-) clusters (Bonferroni-corrected *q*-values were calculated as in *A*). (*C*) As expected from the known mechanism of A3G, mutations found in C- (or G-) coordinated clusters demonstrated higher enrichment relative to nonclustered events (one-tailed Fisher's exact test *P* = 1.35 × 10^−8^). Clustered mutations found in transcribed regions, which have a higher tendency to form ssDNA, exhibit higher enrichment levels compared with all clustered C (or G) mutations (one-tailed Fisher's exact test *P* = 0.045). Fold-change value between different bars is indicated *above* them. (*D*) A strong positive correlation (Pearson's *r* = 0.93, *P* < 0.05) is observed between the level of CCC motif enrichment and the significance threshold used to filter cluster sets (−log[*P*-value]).

Next, we compared the prevalence of C- (or G-) coordinated clusters to that of A- (or T-) coordinated clusters, with C- (or G-) but not A- (or T-) coordinated clusters being potentially a result of A3 activity (on either strand). We observed a significantly higher abundance of C- (or G-) coordinated clusters than A- (or T-) coordinated clusters (243,923 against 210,172—16% higher) (Supplemental Fig. S2) and a higher abundance of C- (or G-) coordinated clusters than expected by chance (Methods; Supplemental Table S1). In order to assess whether a subset of the former clusters were formed by members of the AID/APOBEC family of enzymes, we searched for the A3- or AID-associated mutagenic patterns within the detected C- or G-coordinated clusters while considering the different sequence motif they act on. Namely, we tested the clustered mutations for enrichment in the sequence motifs of the following enzymes: A3B (TC [Hultquist et al. 2011] and TCW [Roberts et al. 2012] motifs; the mutated nucleotide is underlined; W – A or T), A3F (TC [Liddament et al. 2004] and TTC [Liddament et al. 2004; Armitage et al. 2014]), A3G (CC [Hultquist et al. 2011] and CCC [Chelico et al. 2006]) and AID (WRC [Shen and Storb 2004]; R – A or G) by calculating the ratio between the number of mutations falling within the motif to all clustered mutations (e.g., the number of all mutations in the underlined base in CCC divided by the overall number of mutations in C-clusters). Motif enrichment was then determined based on this ratio, following normalization by several similarly calculated backgrounds (Methods). When using the genomic background, we found mutations in the A3G-associated CCC motif to be highly enriched within C- or G-coordinated clusters (Fig. 2A). Additionally, the less stringent of the two A3G motifs, CC, was also found to be enriched, albeit to a lesser extent (Fig. 2A). These findings were robust to the method of normalization or clustering and when considering local variations in nucleotide context (Methods; Supplemental Figs. S3–S6). In contrast, no other APOBEC-related motifs were found to be enriched across clusters by any of the different approaches (Fig. 2A). We further tested all 48 possible trinucleotides that harbor mutated cytosines (i.e., nucleotide triplets with a cytosine in the ancestral state showing a derived mutation in any of the three positions of the triplet) and found, with the exception of CpG-containing triplets, CCC to be the only significantly enriched motif (Fig. 2B; Supplemental Fig. S7).

While a higher mutation rate is expected at CpG dinucleotides due to the spontaneous deamination of 5-methylcytosine (Bird 1980; Sved and Bird 1990), several lines of evidence suggest this effect cannot account for the enrichment of the A3G-associated motif. First, when repeating our analysis with a CpG-filtered mutation set and a CpG-masked genome as a background, we again found an enrichment of the CCC motif in C- and G-coordinated clusters (Supplemental Fig. S8A). Second, the high enrichment value of the A3G motif in the full data set (Fig. 2A) is not restricted to occurrences of a CCCG motif (Supplemental Fig. S8B). Third, we found the CCC motif to contribute additively to mutated cytosines in CCCpG (Supplemental Fig. S8C). In addition, we tested for the enrichment of TTT motifs within T- (or A-) clusters as a negative control for the possibility that CCC enrichment is biased due to any possible mutational effects in homotypic trinucleotide motifs and found no evidence of enrichment (Supplemental Fig. S9).

Since A3G mutagenesis mainly occurs in clusters and is limited to ssDNA, one would expect that mutations within genomic regions with a higher tendency to form a single-stranded intermediate would show increased enrichment of the A3G-associated motif. Transcription is a major physiological cause for the unwinding of genomic double-helical DNA and therefore forms a convenient substrate for A3G (Schumacher et al. 2005). As expected from the biochemical activity of A3G, clustered mutations were found to exhibit elevated motif enrichment in comparison to single point-mutations, while clusters found within transcribed regions were also found to be further enriched with the A3G motif (Fig. 2C).

The strong correlation of CCC motif enrichment and the confidence level of the clusters (*r* = 0.93) (Fig. 2D) demonstrate tight linkage between an overrepresentation of the A3G motif and the occurrence of clustered mutations. Namely, as the probability that a given cluster is a random group of separated point mutations decreases, the level of enrichment of the CCC motif increases, indicating an A3G-specific mutagenesis pattern. Combined, this body of evidence strongly supports that concurrent A3G-induced mutations have contributed to human evolution.

### APOBEC3G-induced genome evolution is common to all hominids

**Figure 3.**
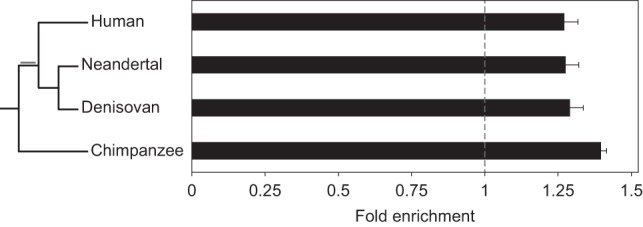
APOBEC3G clustered mutations are observed across all hominid lineages. An analysis identical to that performed on mutations in the human lineage was used to analyze chimpanzee mutations (from the point of divergence from the human lineage) and lineage-specific mutation sets from modern human and the archaic hominins: Denisovan and Neandertal. All sets contain clusters enriched with mutagenic patterns associated with A3G activity (cluster *P* < 0.01; one-tailed Fisher's exact test *P* < 6.8 × 10^−11^). Additionally, mutations common to all three *Homo* lineages (marked in gray on the phylogenetic tree) also showed evidence of enrichment with a value of 1.40 ± 0.03. The lengths of branches in the phylogenetic tree are not drawn to scale. Error bars represent the standard errors that were calculated using a block bootstrap approach.

Our findings indicate that APOBEC3G has contributed to human genome evolution by the introduction of clustered mutations. We next examined whether this phenomenon is limited to the modern human lineage, or—as could be expected by the timing of the APOBEC family expansion—may be widespread across hominids. We applied our approach to test for APOBECs activity in chimpanzee and archaic humans (Neandertal and Denisovan), using recently published sequencing data (Meyer et al. 2012; Prüfer et al. 2014). This allowed us to distinguish between mutations specific to chimpanzee, those arising early in the branch common to both modern and archaic humans after the human-chimpanzee split, and more recent mutations specific to Neandertals, Denisovans, or modern humans (Table 1; Fig. 3). We found that all lineages exhibit an enrichment in the A3G signature within clustered mutations (compared to the genomic background) (Fig. 3) and a tight positive correlation between CCC motif enrichment and the cluster's level of confidence given by their *P*-value (mean *r* = 0.96) (Supplemental Fig. S10). Combined, these results illustrate that A3G-related mutagenesis is a common feature across hominids.

Given that A3G is a primate-specific enzyme, we sought to confirm our results by using a set of nonprimate genomes as a negative control (Keane et al. 2011). A similar analysis performed on a genomic set of mutations called between different mouse strains yielded no enrichment in the CCC motif (Supplemental Fig. S11). Moreover, the mouse A3 motif (TYC; Y – C or T) was also not found to be enriched within clustered mutations (Supplemental Fig. S11). These findings point to APOBEC-induced evolution being a primate-specific phenomenon. This nonprimate control also supports the argument that the effect observed is not the result of nonenzymatic processes, such as spontaneous cytosine deamination or guanine oxidation, which are common to both primates and the species considered for the nonprimate control.

### APOBEC3G activity is overrepresented in functional regions

**Figure 4.**
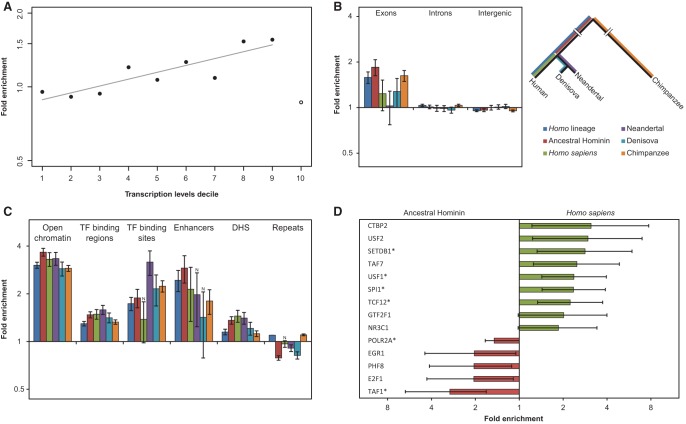
APOBEC3G mutations are associated with transcription and overrepresented in exons and regulatory regions. (*A*) The fold enrichment of the CCC motif in the first nine deciles (solid circles) of the A3G set relative to other mutations is strongly and positively correlated with expression levels (Pearson's correlation coefficient *r* = 0.87, *P* = 0.0012). Notably, the top decile (open circle) behaves differently in terms of fold-change and mutation counts. This can be expected from the increased evolutionary conservation of the most highly transcribed regions, which tend to show a lower tolerance to mutations, let alone clusters of mutations. (*B*) We distinguished between inter-genic regions, introns, and exons. An overrepresentation of A3G mutations within exons relative to the proportion of non-A3G mutations was found in most lineages (*q* < 0.05 for all regions that were tested in *Homo* and *Pan* and both, exons and inter-genic regions in modern and archaic humans. Two-tailed Fisher's exact test with Bonferroni correction). Error bars represent the standard errors that were calculated using a block bootstrap approach. (*C*) Regulatory regions are highly enriched in A3G mutations. Several transcriptional regulatory regions were inspected: open chromatin, transcription factor binding regions and binding sites, enhancers, and DNase I hypersensitive sites. All regions were found to be enriched in most of the lineages (*q* ≤ 0.05, two-tailed Fisher's exact test after Bonferroni correction; N indicates nonsignificance). Error bars represent the standard errors that were calculated using a block bootstrap approach. (*D*) The binding regions of various transcription factors were tested, and several of them showed differential A3G-related mutagenic activity before and after the split of archaic and modern humans. Red bars indicate enrichment before the split, while green bars mark enrichment in the derived lineages after the split. Only results with *P* < 0.05 (two-tailed Fisher's exact test) are shown. (*) FDR-adjusted *P*-value < 0.05. Error bars represent two-sided 95% confidence intervals for the exact test.

We defined the set of potentially A3G-induced mutations as all the mutations within significant C- or G- coordinated clusters (*P* < 0.01) that have at least one CCC mutation (Table 1; Supplemental Tables S2, S3). We found a significant overrepresentation of such potential A3G clusters in all hominid lineages (Supplemental Fig. S12). In order to test for characteristics unique to the set of A3G-induced mutations, we compared the genomic distribution of this set of mutations to a control set composed of all other mutations, independently for each of the lineages. As shown in Figure 2C, clustered mutations within transcribed regions were highly enriched in the A3G motif. This evidence and the fact that transcribed regions have an ssDNA intermediate make it reasonable to expect that actively transcribed regions will be more susceptible to A3G-induced mutagenesis. Indeed, the A3G mutations set includes 1.7-fold more mutations in transcribed regions relative to the control set (Supplemental Fig. S13). Furthermore, a robust correlation was found between transcription levels to the normalized abundance of A3G-induced mutations (Fig. 4A). As genes are the main transcriptional units, we investigated whether our A3G set is enriched with intra-genic mutations. Indeed, exonic (but not intronic or inter-genic) regions were found to be enriched in most of the hominin lineages tested (Fig. 4B). When distinguishing between different exon types, enrichment in coding sequences (CDS) and 5′ untranslated regions (5′ UTR) was detected in several lineages (Supplemental Fig. S14). We next tested whether the enrichment in A3G mutations within CDS has any evolutionary impact in terms of potential altered functionality. In fact, more than a third (51/147) of the A3G-induced mutations in CDS were found to cause amino acid substitutions across 31 genes in the *Homo* lineage alone (Supplemental Table S4). In some cases, a cluster is only partially localized in a CDS, but in other instances the whole A3G-induced cluster, constituting up to five different amino acid substitutions, can be found in the coding sequences of a single exon within the genes: *ERICH3*, *CEMP1*, *MADCAM1*, *PLIN4*, and *IBSP*.

The overrepresentation of A3G mutations in 5′ UTRs led us to hypothesize that A3G mutagenesis could also have functional effects in regulatory regions. Indeed, we found all regulatory elements such as enhancers, transcription factor binding sites, and DNase I hypersensitive regions (DHS) to be enriched for A3G-induced mutations (Fig. 4C). The additive effect between overlapping CCC and CG motifs (Supplemental Fig. S8C) suggests that the A3G-mutations set contains mutations formed by both A3G-induced mutagenesis and nonenzymatic CpG deamination. To rule out that enrichment in functional genomic regions is driven by the latter, we excluded CCCG mutations from the set and obtained comparable results (Supplemental Fig. S15). Next, we focused on transcription factor binding regions (ENCODE ChIP-seq peaks). Interestingly, when comparing ancestral and modern human branches (i.e., before and after the split of modern and archaic humans), several transcription factor binding regions were found to exhibit different tendencies to accumulate A3G mutations between ancestral and derived branches (Fig. 4D). Namely, we found that some of the binding regions were significantly enriched in A3G mutagenesis in early human evolution while others showed a significant increase in A3G activity only in modern humans. Notably, this provides evidence of a shifting landscape of A3G activity along different regions of the genome at different evolutionary time periods and suggests that A3G may have played a role in the evolution of functional regulatory differences among hominids (Fig. 4D).

In conclusion, after carefully controlling for possible confounders, C- (and G-) coordinated clusters across hominids show an enrichment of the hallmark signature of A3G mutagenesis, suggesting that its activity may have been responsible for the introduction of tens of thousands of mutations with unique features in hominid evolution and probably many more in the entire primate tree. Specifically, these mutations are localized in transcribed regions, especially exons, and in regulatory elements, where they show signs of varying rates of occurrence at different evolutionary time periods. Genomic changes in both regions individually could have contributed to the evolution of phenotypes of both modern humans and closely related species.

## Discussion

In this study, we performed a comprehensive analysis of mutagenesis patterns in the different branches of the hominid phylogenetic tree. By applying an unbiased approach to define clusters of mutations and to examine different APOBEC- and non-APOBEC-related motifs, we found extensive evidence for specific A3G activity on each of the examined branches. Beyond the detection of an increased number of clusters of mutations due to cytosine deamination, several observations provide strong support for the conclusion, including the overrepresentation of the A3G-associated CCC motif within clustered mutations relative to different backgrounds, a strong correlation between the reliability of observing a cluster and the proportion of the effect attributed to A3G motif, and the high tendency for A3G mutagenesis to coincide with transcriptional activity. To the best of our knowledge, these findings are the first evidence for site-directed, enzyme-induced evolution catalyzed by A3G. This activity bears the hallmark signature of mutations catalyzed by A3G, a protein with specific and potent mutagenic properties. As A3G mutagenic activity introduces more diversity than a single point mutation, it can potentially play a greater role in fueling the evolution of novel function. Together, these results provide strong evidence supporting a role for A3G in contributing to the divergence of hominids in a manner likely to have shaped functional differences among primates.

A3G is a member of the A3 family, which evolved due to selective pressures imposed by the expansion of primate-specific retroviruses and retroelements (Sawyer et al. 2004; Zhang and Webb 2004). While viruses generally infect somatic cells, retroelement integration is mainly destructive when it occurs in the germline. Indeed, A3 members are known to be expressed in the reproductive system, and A3G exhibits the highest expression levels among the seven paralogs in testis and testis germ cells, and the second highest expression levels in ovary (Su et al. 2004; Koning et al. 2009; Refsland et al. 2010; Burns et al. 2013b). Specifically, the expression of A3G in testis germ cells was shown using several different techniques, such as DNA microarrays (Su et al. 2004), RNA-seq, and protein immunohistochemistry (Uhlen et al. 2015). Another support for the expression of A3G in germline comes from the recently published evidence for the inherited A3G-dependent diversification of retroelements along primate evolution (Knisbacher and Levanon 2016). The prevailing notion of A3G being maintained under strict control, limiting its localization to the cytoplasm, has been brought into question by several studies that demonstrated it can be partially localized to the nuclear compartment (Stopak et al. 2003; Hill et al. 2006; Depboylu et al. 2007). It has also been shown that A3G can be recruited to double-strand breaks in genomic DNA (Nowarski et al. 2012) and that it may shuttle to the nucleus in response against LINE-1 retrotransposition (Kinomoto et al. 2007). A3G expression in the germline and its small nuclear fraction, both fundamental conditions for inherited mutagenesis, provide a window of opportunity for the contribution of A3G to genome evolution. Our findings support that A3G, originally an anti-viral agent, has indeed acquired the capacity of contributing to genome diversity.

In terms of evolutionary innovation, the ability of A3G to introduce genomic germline mutations might provide different advantages in comparison to random single-nucleotide substitutions. The generation of adjacent mutations by spatiotemporally separated stochastic events is time-consuming and can also be limited by particular features of the fitness landscape when successive steps limit its traversal. In contrast, A3G can form clusters of closely located concurrent mutations in a single molecular event, instantly providing a mechanism that can prove advantageous when multiple mutations are required for increase in fitness. Yet, as any other mutation, A3G-related mutations are subjected to natural selection or drift. However, the higher efficacy of A3G to introduce mutations in highly transcribed regions and regulatory elements increases the likelihood that these types of concurrent mutations have functional consequences and that A3G mutagenesis may have played a role in the evolution of phenotypic differences during primate evolution.

While the lineages tested in this work are all members of the hominid family, the conclusions should not be limited to this small set of the primate clade. Rather, this study can be considered as a case study that provides evidence for a novel mechanism in primate evolution.

A3G-related mutagenesis adds one additional mechanism to the variety of previously known processes contributing to species divergence. While A3G contributes a relatively small fraction of the total number of mutations, it nevertheless tends to act in a functionally enriched fraction of the genome, on account of the higher tendency of these regions to form ssDNA intermediates which A3G targets. Therefore, A3G-related mutations are, on average, more likely to be targeted by natural selection, either positive or negative. Additionally, the approach of limiting our analysis to clusters of coordinated mutations is rather conservative. While the method helps ensure specificity in the detection of the desired signal, the exclusion of clusters containing other types of mutations necessarily leads to reduced sensitivity in the detection of the complete set of A3G-induced events. Other A3G-induced events, where linkage disequilibrium has broken down the number of observed concurrent mutations, are also not captured by our method. Hence, it is important to consider the numbers reported here as a conservative estimate of the contribution of A3G activity to primate divergence. Another limitation of our approach is the identification of clusters composed of more distantly spread concurrent mutations (as may be expected from the activity of other genes in the APOBEC family, e.g., APOBEC3B [Roberts et al. 2012; Burns et al. 2013b]), which is due to the increased chance that other mutations interrupt longer coordinated clusters. An additional feature of the A3G mutagenesis pattern is its higher frequency of C-to-T substitutions relative to C-to-G or C-to-A. This pattern is different from the one previously published for A3B in cancer models in which similar frequencies of C-to-T and C-to-G were observed (Roberts et al. 2012, 2013). Hence, while A3G seems to have a primary role, we do not rule out the possibility that other APOBEC family members may have also contributed mutations to the examined genomes.

In this study, we focused on the most established A3G motifs, CC and CCC. Several studies that investigated the anti-viral activity of A3G examined the tetranucleotide motif and found various preferred 3′ nucleotides relative to the mutated cytosines (Harris et al. 2003; Liddament et al. 2004; Armitage et al. 2014). Our results regarding the tetranucleotide motifs vary from the published patterns mainly in the higher abundance of CCCG, which might be a result of spontaneous CpG deamination events that accumulated during evolution.

This novel ability of A3G to induce inherited mutations opens up a realm of possibilities for other interesting avenues of research, some with potentially novel medical implications. While little is still known, it is of interest to understand if there are conditions that can modulate A3G activity causing mutations on heritable material. Whether there are direct functional, genetic, or environmental drivers, or it is simply a side effect of errors in the control of its compartmentalization within the cell or in its activity, are all open questions. Among possible conditions, we know little about its relation to possible historical factors, such as whether periods of increased exposure to viruses or peaks in the activity of retroelements may have contributed to the detected patterns. It is well known that A3G activity is also induced in response to HIV (Hultquist et al. 2011). It would thus also be interesting to understand if certain types of viral infection or their occurrence during particular cellular or developmental stages may increase the potential for heritable induced A3G mutagenesis, or whether additional factors such as frequency and timing of A3G nuclear activity may influence its accessibility to different types of genomic regions and their associated functions. In addition, further work examining the role of A3G in divergence and polymorphism within the human population would be of a great interest.

## Methods

### Calling mutations

Lineage-specific substitutions in human were computed by parsimony-based approach using alignments from the UCSC Genome Browser for the human reference (hg19) and three outgroups: chimpanzee (panTro2), orangutan (ponAbe2), and rhesus macaque (rheMac2) (Kent et al. 2002). Sites where the chimpanzee allele was available (see “filtering” below) and its state was confirmed by matching that in either orangutan or rhesus macaque were kept for further analysis. In these cases, the chimpanzee allele was assigned as the ancestral human state, and human divergences were then called for positions where the human reference was different from the inferred ancestral state.

For filtering, we labeled as “missing” all positions for each reference genome individually, falling in an alignment gap, with an ambiguous allele (e.g., “N”), or falling within a region of poor synteny with human (as determined by the UCSC human/chimpanzee, human/orangutan, or human/rhesus macaque syntenic nets). Positions in the alignment where human or chimpanzee, or where both orangutan and rhesus macaque, were missing were filtered out from our data set. Mutations within simple and low-complexity repeats and segmental duplications were filtered out due to their low reliability (Jurka 2000). For all human/chimpanzee syntenic regions, the Altai Neandertal and Denisovan states were determined using the sequencing data of Prüfer et al. (2014) and Meyer et al. (2012), respectively, and filtered as described in Prüfer et al. (2014). Using the inferred human/chimpanzee ancestral state, we distinguished between mutations occurring before and after the split of modern and archaic human populations. Genomic variation data for different mouse strains was obtained from Keane et al. (2011). Mutations specific to the C57BL/6J mouse strain (mm9 reference genome) were called as previously described using the NZO/HlLtJ, NOD/ShiLtJ, and A/J strains as outgroups. Mutation calls performed using these strains resulted in a set of mutations which is comparable in size to that of the tested hominid branches (∼2.6M mutations) (Table 1). Additionally, these strains, while sufficiently distant on an evolutionary scale to provide similar numbers of mutations as hominids, are sufficiently close as to ensure a similarly low likelihood for multiple mutations at a site, making them effectively comparable under the parsimony-based approach utilized in our pipeline.

### Clustering

Mutations were clustered based on previously published methodology (Roberts et al. 2012, 2013) with a single modification where clusters were defined as a group of at least two mutations that are separated by 50 bp or less from each other. Clusters were named by the ancestral nucleotide state on which the mutation arose (e.g., clusters composed solely from mutation derived from cytosines were classified as C-coordinated clusters) (see Supplemental Data). Clusters composed of mutations originating from different ancestral nucleotide states were classified as N-clusters. The interval between pairs of mutations was limited to 50 bp in order to allow a sufficient recovery of non-N clusters, when taking into account the large number of point mutations generated along the evolution of primate species and the high probability of a random recent substitution to disturb a previously formed long coordinated cluster. The same clustering methodology was used in Supplemental Figure S6, except the maximal distance between mutations were set to either 100, 300, or 1000 bp. Cluster *P*-values were computed using the negative binomial distribution:

$$p=\overset{x-k}{\underset{\;j=0}{\sum }}\left(\begin{array}{c} \left(k-1)+(\;j-1\right)\\ j \end{array}\right){\left(1-\pi \right)}^{j}\pi^{k-1}.$$

A cluster *P*-value was defined as the probability of observing *k* − 1 mutations in *x* − 1 bp, where *x* denotes the size of the mutation cluster, *k* denotes the number of mutations observed in a cluster, and π denotes the probability of finding a mutation at any random location in the genome.

### Motif enrichment

We tested for an enrichment in APOBEC mutagenesis patterns within mutation clusters. Both positive and negative strands were tested for each motif using its reverse complement sequence strands (e.g., to detect CCC motif in the negative strand, we tested for the GGG motif within G-coordinated clusters). Motif-associated signals were estimated as the fraction of clustered mutations falling in a certain motif out of the total number of clustered mutations. For example, for the CCC motif we estimate the signal as

$$\frac{{\text{Clustered mutations}}_{\;CC\underline{C}}}{{\text{Clustered mutations}\;}_{\underline{C}}},$$

where all clustered mutations within C-coordinated clusters originate from ancestral cytosines by definition. We then compared the original signal against several distinct backgrounds to calculate enrichment $E=\frac{\mathrm{Signal}}{\mathrm{Background}}$:

*Genomic background*: The original signal compared against the ratio between the number of occurrences of a motif in the genome to the occurrences of the ancestral nucleotide in the genome, e.g., $\frac{{\text{Genome}}_{CCC}}{{\text{Genome}}_{C}}$. Therefore the enrichment is defined as:

$$E=\frac{\;\frac{{\text{Clustered mutations}}_{\;CC\underline{C}}}{{\text{Clustered mutations}}_{\;\underline{C}}}\;}{\frac{{\text{Genome}}_{\;CCC}}{{\text{Genome}}_{\;C}}}.$$

*Random sets of mutations*: For each set of mutations, we generated 100 random sets of mutations as an arbitrary control. Each set was of the same size and with the same mutation types as the original set. Namely, for each mutation in the original set we randomized its genomic location using BEDTools shuffle command (Quinlan and Hall 2010). We then compared the original signal against the fraction of mutations falling within motifs out of the total number of clustered mutations for all 100 sets, e.g., $\frac{{\text{Randomized clustered mutations}}_{\;CC\underline{C}}}{{\text{Randomized clustered mutations}}_{\;\underline{C}}}$. Therefore, the enrichment is defined as:

$$E=\frac{\frac{{\text{Clustered mutations}}_{\;CC\underline{C}}}{{\text{Clustered mutations}}_{\;\underline{C}}}}{\;\frac{{\text{Randomized clustered mutations}}_{\;CC\underline{C}}}{{\text{Randomized clustered mutations}}_{\;\underline{C}}}}.$$

*Random sets of clusters*: For each set of clusters, we generated 100 sets of random clusters. Each set included the same size of clusters from each type, with the same cluster length but in random genomic loci. In addition, the random clusters preserve the same number of mutations, with the same mutation types and the inner mutations' distances from clusters boundaries. Then, we calculated a ratio similar to the one that was computed for random mutations sets.

*Local nucleotide context*: We calculated the ratio between the number of occurrences of a motif in the 10-kb region centered on the middle of each cluster to the occurrences of the ancestral nucleotide in the same context, e.g., $\frac{{\text{Cluster context}}_{\;CCC}}{{\text{Cluster context}}_{\;C}}$. Therefore the enrichment is defined as:

$$E=\frac{\;\frac{{\text{Clustered mutations}}_{\;CC\underline{C}}\;}{{\text{Clustered mutations}}_{\;\underline{C}}}}{\frac{{\text{Cluster context}}_{\;CCC}}{{\text{Cluster context}}_{\;C}}}.$$

### Calculating the expected number of clusters

The expected number of C- (or G-) coordinated clusters was calculated as the probability that all mutations in a given cluster originated from the same ancestral nucleotide using the prevalence of mutations derived from each ancestral state: $\text{Expected}=\overset{k}{\underset{i=2}{\sum }}(N_{i}\cdot p^{i})$, where *k* is the maximal number of mutations in a single cluster, *N*_*i*_ is the number of clusters with *i* mutations, and *p* is the prevalence of mutation from a specific ancestral state.

### Definition of A3G clusters

A3G clusters were defined as C- (or G-) coordinated clusters with at least one CCC (or GGG) mutation. The expected number of A3G clusters was calculated by the probability of having at least one CCC mutation in a cluster:

$$\text{Expected}=\overset{k}{\underset{i=2}{\sum }}\left(N_{i}\overset{i}{\underset{\;j=1}{\sum }}\left(\begin{array}{c} n\\ j \end{array}\right)p^{j}{\left(1-p\right)}^{n-j}\right),$$

where *p* is the probability of mutation falling within a motif, *k* is the maximal cluster size, and *N*_*i*_ is the total number of clusters with *i* mutations.

### Error estimation

Standard errors were calculated using a block bootstrap approach (Kunsch 1989; Liu and Singh 1992; Lahiri 2003; Keinan et al. 2007). Resampling was carried out by dividing the genome into 35,165 equal-sized blocks of ∼90 kb. The blocks were then randomly sampled with replacement to create 10,000 bootstrap genome samples.

### Functional regions

A3G-induced sets of mutations were defined as all mutations found within A3G clusters. The fold-change for mutations in different genomic regions between A3G-induced mutations to all other mutations (control set) was computed as the ratio between the fraction of mutations falling in a given region relative to those in the control set. The enrichment in a specific functional region was calculated as follows:

$$\frac{\frac{{\text{mutations in a functional region}}_{A3G\;\mathrm{set}}}{{\text{mutations}}_{A3G\;\mathrm{set}}}}{\frac{{\text{mutations in a functional region}}_{\mathrm{Control}\;\mathrm{set}}}{{\mathrm{mutations}}_{\mathrm{Control}\;\mathrm{set}}}}.$$

Transcribed regions and open chromatin data from H1-hESC, DHS peaks, and chromatin immunoprecipitation (ChIP)-seq peaks from ESC were used as transcription factor binding regions and were obtained from the ENCODE Project (Rosenbloom et al. 2013). The respective ENCODE tables—H1-hESC (under transcription track, for transcription levels data), wgEncodeRegTfbsClusteredV3 (under Txn Factor ChIP track, for TF binding regions data) and H1-hESC Syn Pk (under Open Chrom Synth track for open chromatin data)—were downloaded from the UCSC Table Browser (Karolchik et al. 2004). Transcription factor binding site data were obtained from factorbook version 3 (Wang et al. 2013). CpG islands data were retrieved from the UCSC Table Browser (Karolchik et al. 2004). Data for the location of transcriptional enhancers were downloaded from the ORegAnno database, picking entries annotated as regulatory regions (Griffith et al. 2007). To ensure that the enrichment in genes is not due to A3G activity in the retrotransposition phase of processed pseudogenes, we computed the number of *Homo* lineage A3G mutations within processed pseudogenes and found negligible contribution (83 out of 13,011 mutations). Processed pseudogenes data were downloaded from the UCSC Table Browser (Karolchik et al. 2004).
